# The Story of the Insane from Year to Year

**Published:** 1890-11-08

**Authors:** 


### THE STORY OF THE INSANE FROM YEAR TO YEAR.

### WILTS COUNTY ASYLUM, DEVIZES.

The Wilts County Council have passed a resolution that no pension be given by the Council to any of its officers, and they have recommended the Asylum Committee not to grant pensions to any asylum official. The visitors say they have made all engagements subsequent to January 1st, 1890, on these terms, but they somewhat illogically add that, in view of the Local Government Board circular of August 17th, they entertain some doubt as to whether or no their power of granting pensions has been taken from them by the County Council resolution. Manifestly they can still vote the pension, but it cannot be paid until confirmed by the Council. One thing is quite certain—namely, the abolishing of pensions will have a most injurious effect on the asylum, and, moreover, it is an extremely expensive move on the part of the Council. There is no doubt that an asylum officer, or attendant, or nurse, becomes, about the age of 55, quite unfit for duty, and if retained in the service deputies must be provided, and as it is utterly impossible for the attendant to save enough money to keep him in his old age out of £30 or £40 a-year, or the nurse out of £15 or £20, it is clear that the wages must be increased all round, alike to the deserving and undeserving. Now there are three courses open to the visitors and Council—first, to act upon the pension clause of the Lunacy Act; second, to dismiss ths official when he reaches 55 years of age or so without a pension, and without provision for his declining years; or third, they can increase the wages and salaries all round, and so enable the official to save money. Whatever may be said against pensions theoretically, they act well in asylums, and are always to be recommended. Perhaps no Committee of Visitors is bold enough or inhuman enough to dismiss an old official, so he would be allowed to stay on, and a deputy would be engaged at a greater cost than the pension of the old official would have amounted to. If the wages be increased all round, then the sum of extra money which the county would have to find would be enormous. It seems from the experience of other asylums that at any given time only about one in ten of the officials are likely to claim pensions, and that the average duration of life subsequent to the pension is only some seven or eight years. Increasing wages and salaries in the Devizes Asylum would, therefore, mean the squandering of many hundred pounds annually. We have entered more fully than usual into this question, because we look upon the certainty of pensions as one of the things on which the future of our County Asylums depends, and because we are vain enough to hope that if these words should fall into the hands of the Wilts County Council they may induce them to reconsider their decision. The asylum contained 681 patients on the last day of this year, and during a part of the year the building was overcrowded in some wards. Additional dormitories have been built for fifty patients, but this must be a mere makeshift, and much more must be done soon, or the visitors will find themselves compelled to board out these patients in some other asylum at 14s. a-week. The visitors say that "the anxious, careful, and kindly manner in which the medical superintendent invariably performs his duties meets with the entire approbation of the committee." Mr. Bowes' report is, as usual, clear and complete—a fair model of what such documents should be. We learn that eight of the fifty-nine deaths were owing to phthisis. Employment of the patients seems well carried out. Seventy-four per cent. of the men and 58 per cent. of the women do something useful. We are pleased to note that the weekly rate of maintenance has risen from 7s. to 7s. 7d.

